# Sleep quality among inpatients of Spanish public hospitals

**DOI:** 10.1038/s41598-022-26412-7

**Published:** 2022-12-20

**Authors:** Filip Bellon, Robyn Stremler, Esther Rubinat-Arnaldo, Julia M. Padilla-Martínez, Elvira Casado-Ramirez, Montserrat Sánchez-Ortuño, Montserrat Gea-Sánchez, Yolanda Martin-Vaquero, Teresa Moreno-Casbas, Eva Abad-Corpa

**Affiliations:** 1 GESEC Group, Department of Nursing and Physiotherapy, Faculty of Nursing and Physiotherapy, University of Lleida, Montserrat Roig, 25198 Lleida, Spain; 2 Healthcare Research Group (GRECS), Institute of Biomedical Research Lleida (IRBLleida), Av. Alcalde Rovira Roure, 80, 25198 Lleida, Spain; 3 Lawrence S. Bloomberg Faculty of Nursing, University of Toronto, Toronto, ON Canada; 4 The Hospital for Sick Children (SickKids), Toronto, ON Canada; 5 Center for Biomedical Research on Diabetes and Associated Metabolic Diseases (CIBERDEM), Instituto de Salud Carlos III, Barcelona, Spain; 6 University of Murcia-Murcia Health Service (IMIB-Arrixaca), Campus Universitario, 1, 30100 Murcia, Spain; 7 Nursing and Healthcare Research Unit (Investén-Isciii), Av. Monforte de Lemos, 5. Pabellón 13, 28029 Madrid, Spain; 8 Biomedical Research Center for Fragility and Healthy Aging (CIBERFES), Av. Monforte de Lemos, 5. Pabellón 11, 28029 Madrid, Spain; 9 University of Murcia, Campus Universitario, 1, 30100 Murcia, Spain; 10 Zamora Healthcare Complex, Zamora Health Care Management, Av. de Requejo, 35, 49022 Zamora, Spain

**Keywords:** Health care, Health occupations, Medical research

## Abstract

Sleep is a complex process and is needed both in health and illness. Deprivation of sleep is known to have multiple negative physiological effects on people’s bodies and minds. Despite the awareness of these harmful effects, previous studies have shown that sleep is poor among hospitalised patients. We utilized an observational design with 343 patients recruited from medical and surgical units in 12 hospitals located in nine Spanish regions. Sociodemographic and clinical characteristics of patients were collected. Sleep quality at admission and during hospitalisation was measured by the Pittsburgh Sleep Quality Index. Sleep quantity was self-reported by patients in hours and minutes. Mean PSQI score before and during hospitalisation were respectively 8.62 ± 4.49 and 11.31 ± 4.04. Also, inpatients slept about an hour less during their hospital stay. Lower educational level, sedative medication intake, and multi-morbidity was shown to be associated with poorer sleep quality during hospitalisation. A higher level of habitual physical activity has shown to correlate positively with sleep quality in hospital. Our study showed poor sleep quality and quantity of inpatients and a drastic deterioration of sleep in hospital versus at home. These results may be helpful in drawing attention to patients’ sleep in hospitals and encouraging interventions to improve sleep.

## Introduction

Sleep, essential for healthy functioning, is characterized by sensory disconnection^1^. Buysse et al.^2^ defined sleep as “a multidimensional pattern of sleep-wakefulness, adapted to individual, social and environmental demands, that promotes physical and mental well-being”. Without question, sleep has a global restorative function for the organism, fundamental for maintaining neurobehavioral activity and brain health during wakefulness^1,3^.

Sleep quality and quantity have a critical role in promoting health^4^, and poor sleep quality has been associated with an increased risk of cardiovascular events^5^, cancer^6^, metabolic disorder^7^, and all-cause mortality^8^. Moreover, chronic poor sleep has been related to cognitive function impairment and neurodegenerative diseases^9^, a weakened immune system^10,11^ and a higher fall risk^12^. Additionally, it has been demonstrated that good sleep quality can be of therapeutic use for patients with chronic pain^13^, or mental disorders^14^.

There are numerous patient-related factors that have potential negative impact on sleep of patients such as pain, the underlying acute illness, unemployment or age, and psychological factors such as anxiety^15–18^. A review on sleep health concluded that sociodemographic variables are of enormous importance when evaluating sleep. Factors such as low physical activity or high fat diets can impair sleep, and an increased incidence of sleep disruptions can also be attributed to the female gender and low socioeconomic level^19^. Additionally, previous studies have suggested that patients’ sleep quality and quantity during hospitalisation is poor^20–23^, and associated sleep disturbances, such as a decrease in total sleep time, poor sleep efficiency, increased awakenings per night, or an increased sleep onset latency, in both domains of self-reported sleep quality and objective measured sleep, are shown to continue up to 12 months after hospitalisation^24^. During hospitalisation, symptoms such as abnormal melatonin secretion, showed by both a delay in the endogenous rhythm of plasma melatonin and 6-SMT excretion in urine^25,26^, or sleep beathing disorders or obstructive sleep apnea^27,28^ have been observed. The clinical environment may be a cause of this sleep impairment^20^. Factors such as light and sound disturbances, night-time care interventions, environmental temperature, and bed comfort are possible factors that can lead to poor sleep, causing sleep disruptions during the night and inducing misalignment in the sleep–wake cycles^23,29^. However, as hospitals are a place of healing, it could be expected that they should provide the best possible conditions to achieve this healing process. Also, hospitals could have an educational function by offering exemplary behaviour and support to patients in order to achieve sleep health in the long term, even after hospitalisation^30^.

To date, in Spain, no previous multicentre studies, including patients from public hospitals from several regions, have been carried out in medical and surgical units to describe sleep quality and quantity.

Therefore, we aimed:

- To assess self-reported sleep quality and quantity of medical and surgical unit inpatients before and during their hospitalisation.
- To describe the association of inpatients’ sociodemographic and poor health habits and sedentary behavior to their sleep quality

## Methods

### Study design and participants

**Figure 1:**
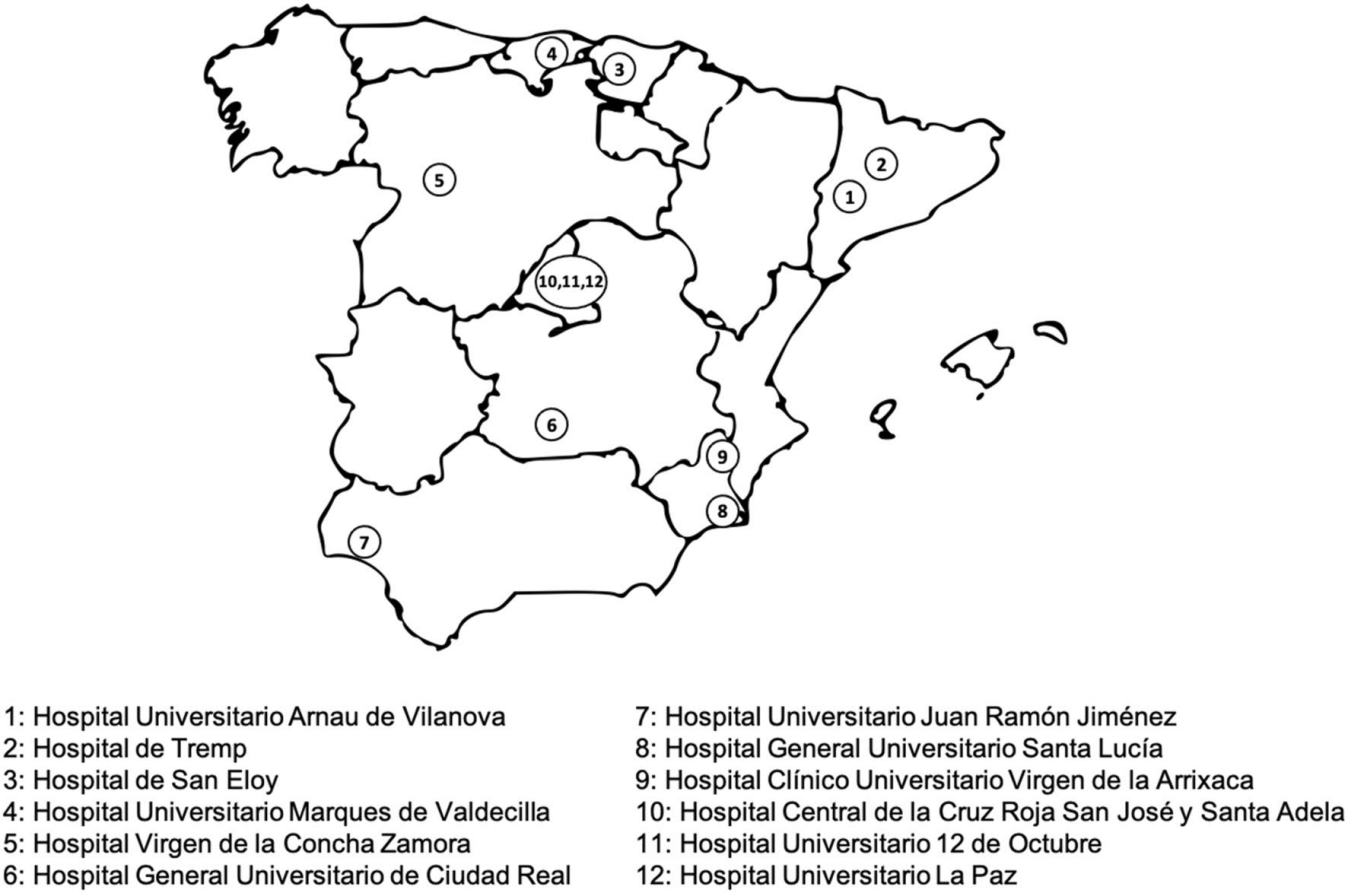
Geographical distribution of included hospitals.

A multicentre, observational descriptive study was carried out, recruiting patients from medical and surgical units in 12 hospitals of the Spanish National Health System located in nine Spanish regions (Fig. 1). All hospitals were participants of the SueñOn ® health awareness initiative^31^. Inpatients, aged 18 years or older with at least 4 consecutive nights of hospitalisation who consented to participate voluntarily in the project were included in this study. Patients with visual or auditory impairment, intellectual disability or moderate to severe cognitive impairment as reflected in the clinical patients’ record were excluded from this study. We did not include patients with a COVID-19 infection.

Data were collected between January 2020 to January 2022 and patients were included on pre-established days every month. The Strengthening the Reporting of Observational Studies in Epidemiology (STROBE) guideline was followed for reporting this study.

### Data collection

#### Sociodemographic and clinical characteristics

Data were collected on age, gender, education level, current work situation, mean income per household (euros per month), Body Mass Index (BMI), and unhealthy habits such as smoking or any other recreational drug intake.

Patients’ clinical characteristics were collected by the nursing staff of the unit. The motive of admission was classified and data on diagnosed and self-reported comorbidities of the patient were collected. Patients were classified as having multimorbidity if multiple diseases or conditions were reported using a definition of two or more conditions^32^. Also, covariates such as sedative hypnotic medication use, and staying in a single or shared room were recorded.

To assess physical activity level over the last seven days before hospitalisation, the International Physical Activity Questionnaire (IPAQ)^33^ was used.

#### Sleep quality and quantity

The Spanish version of the Pittsburgh Sleep Quality Index^34^ (PSQI) was used to assess patients’ perception of sleep quality referring to the past 1 month, and at discharge referring to the patients’ hospital stay. The PSQI is a validated and highly reliable self-report questionnaire consisting of 19 questions divided into seven major components. These seven components are: subjective quality of sleep, sleep latency, length of sleep, sleep efficiency, sleep disturbances, use of sleep medication and daytime dysfunction. Scores range from 0 to 3 for each component and the global score ranging from 0 to 21 is obtained by the sum of all components scores. A higher global PSQI score indicates worse sleep quality^2^. Using a cut-of score of 5 to differentiate poor sleep with good sleep the PSQI reported a sensitivity of 89.6% and a specificity of 86.5%. The validated Spanish version of the PSQI has shown a correlation coefficient of 0.773 for the global score^34^. The question used to evaluate sleep quantity was: “How many hours do you estimate you actually slept each night?”.

### Data analysis

Data were collected on paper and entered into an online REDCap electronic database^35^. Analysis of data was performed using SPSS 25.0. Descriptive data were categorized and compared with study variables. Categorical variables were summarised by percentages and continuous variables were reflected by means and SD. Kolmogorov–Smirnov test was used for checking whether sleep quality and quantity could be assumed to be normally distributed. For skewed or non-normality data non-parametric analysis were performed. Sleep quality and quantity differences at admission and during hospitalization were compared with student’s paired *t* test (Wilcoxon test) and differences between groups were analysed with the student’s *t* test (Mann–Whitney test) or ANOVA (Kruskal–Wallis test). Correlation of physical activity level measured with the IPAQ questionnaire was calculated with the Spearman Rho test. All tests were two-tailed at 0.05 significance level.

### Ethical approval

Patients participated voluntarily and were included in this study after giving their written consent in the informed consent document in regulation with the law 14/2007 on biomedical research and ethical principles after the declaration of Helsinki. Patients were previously informed of the entire process, and their possibility to revoke their participation at any time. Approval of all ethical committees of all participating hospitals were obtained as well as the approval of the ethical committee of the Carlos III health institute (HIP CI CEI PI 18_2019-V3). Additionally, this study was registered with Clinicaltrials.gov (#NCT04113876).

## Results

### General characteristics of the participants

A total of 343 patients from 12 different Spanish public hospitals with a minimum hospital stay of 4 days were included in our study. Our study included hospitals of both rural and urban regions and covered hospitals from geographical areas all over Spain. The mean age of the participants was 63.47 ± 14.96 years. Our sample was comprised of 143 (41.69%) female, and 199 (58.02%) male participants (one patient didn’t identified their sex) and 20.41% of the participants reported being habitual smokers. Included patients were hospitalised in surgical n = 162 (47.23%) and medical n = 181 (52.77%) units. The majority of the patients were retired n = 202 (58.89%), 97 (28.28%) were employed, 37(10.79%) were unemployed, and 2 (0.58%) were students. Over half of our sample (54.52%) had been educated only to the primary level, and only 9.62% were university graduates. Household incomes per capita were mostly (38.38%) between 0 and 1000 euros, 35.86% had an average income between 1001 and 2000 euros monthly, and 9.33% of the participants disposed of > 2000 euros a month (9.33% of the participants did not want to provide data about their income). The mean BMI of the participants was 27.70 ± 5.98. Most patients were diagnosed with multimorbidity (62.09%). Main hospital admission motives were digestive diseases (27.41%), neoplasms (25.07%) and respiratory diseases (20.41%). Most of the patients stayed in double rooms n = 289 (84.26%) and the median hospital stay was 8 days (IQR = 7.00–11.00). More details about sociodemographic variables, hospital admission motive, comorbidities, and the distribution of the 343 participants and missing data are shown in Table 1.

**Table 1:** Sociodemographic characteristics and admission motive of participants.

Total sample	343	%
**Age (mean ± SD)**	63.47 ± 14.69	
< 30	9	2.62
31–50	48	13.99
51–70	159	46.36
> 70	127	37.03
**Unit**		
Surgical unit	162	47.23
Medical unit	181	52.77
**Gender**		
Male	199	58.02
Female	143	41.69
Missing data	1	0.29
**Hospital stay (Median [IQR])**	8.00 [7.00–11.00]	
≤ 7 days	123	35.86
> 7 days	176	51.31
Missing data	44	12.82
**Educational level**		
Primary	187	54.52
Secondary	112	32.65
University	33	9.62
Missing data	11	3.21
**Working situation**		0.00
Working	97	28.28
Unemployed	37	10.79
Retired	202	58.89
Student	2	0.58
Missing data	5	1.46
**Income (euro/month)**		
0–500	29	8.45
501–1000	103	30.03
1001–2000	123	35.86
> 2000	32	9.33
Missing data	54	15.74
**Room type**		
Single	54	15.74
Double	289	84.26
**BMI (mean ± SD)**	27.70 ± 5.98	
< 18.5	5	1.46
18.5 to < 25	117	34.11
25 to < 30	122	35.57
30 or more	96	27.99
Missing data	3	0.87
**Smoker**	70	20.41
**Drug consumer**	10	2.92
**Multimorbity**		
Yes	213	62.09
No	78	22.74
Missing data	52	15.16
**Hospital admission motive**		
Neoplasms	86	25.07
Blood and immunological diseases	12	3.50
Endocrine diseases	16	4.66
Cardiovascular diseases	45	13.12
Respiratory diseases	70	20.41
Digestive diseases	94	27.41
Skin diseases	10	2.92
Musculoskeletal diseases	36	10.50
Genito-urinary diseases	27	7.87

### Sleep quantity and quality before and during hospitalisation

Looking at total sleep quantity, participants’ report of how many hours they actually slept before and during hospitalisation resulted in a mean total sleep time of 56 min less during hospitalisation (6.28 ± 1.64 vs 5.34 ± 1.58, *p* < 0.001).

Global PSQI scores measuring overall sleep quality could be calculated in only 298 patients (86.88%) because of missing data. However, because missing data was sporadic across the 19 questions, we also calculated the scores of the seven PSQI components relating to sleep quality.

Poor sleep quality (PSQI > 5) before hospitalisation was present in 77.00% and was 95.10% during hospitalisation. Mean global PSQI score before hospitalisation was 8.62 ± 4.49 and rose to 11.31 ± 4.04 during hospitalisation showing a decrease in overall sleep quality during hospitalisation compared to sleep quality before admission in the hospital (Z = −8.539, *p* < 0.001). Daytime dysfunction was the lowest scoring component of the PSQI (0.67 ± 0.88) and sleep latency was the highest (1.52 ± 1.09) before hospitalisation. When participants answered the PSQI at discharge, daytime dysfunction remained the lowest component (1.12 ± 1.01), and sleep latency was scored the highest (1.98 ± 1.03), along with sleep duration (1.98 ± 0.94), and sleep efficiency (1.98 ± 1.18). The greatest increases could be seen also in these components; sleep efficiency (+ 0.63, *p* < 0.001), sleep duration (+ 0.54, *p* < 0.001), and sleep latency (+ 0,46, *p* < 0.001) comparing PSQI scores before and during hospitalisation suggesting that these components had the greatest influence on the decline of patients’ sleep quality in the hospital. Student’s *t*-test and Mann–Whitney test confirmed increases in all seven components, all statistically significant except for component 1: subjective sleep quality (*p* = 0.056). Changes in PSQI scores and reported sleep quantity are summarized in Table 2.

**Table 2:** Sleep quality and quantity at admission and during hospitalisation.

	At admission			During hospitalisation			Test and (*p* value)*
	n	Mean	SD	n	Mean	SD	
**Sleep quality component**							
1. Subjective sleep quality	339	1.42	0.89	339	1.54	0.82	Z = −1.915 (0.056)
2. Sleep latency	335	1.52	1.09	335	1.98	1.03	Z = −6.282 (< 0.001)
3. Sleep duration	338	1.44	1.00	338	1.98	0.94	Z = −7.707 (< 0.001)
4. Sleep efficiency	332	1.35	1.22	332	1.98	1.18	Z = −7.128 (< 0.001)
5. Sleep disturbances	316	1.27	0.60	316	1.47	0.60	Z = −4.606 (< 0.001)
6. Sleeping medication	342	1.00	1.34	342	1.28	1.49	Z = −2.887 (0.04)
7. Daytime dysfunction	334	0.67	0.88	334	1.12	1.01	Z = −6.595 (< 0.001)
Global PSQI score	298	8.62	4.49	298	11.31	4.04	Z = −8.539 (< 0.001)
Sleep quantity (h)	338	6.28	1.64	338	5.34	1.58	Z = −8.411 (< 0.001)

*SD* standard deviation

*Student’s paired t-test or Wilcoxon test.

### Association of patients’ sociodemographic and clinical characteristics with sleep quality and quantity

A decrease in sleep quality could be observed in all sociodemographic categories during hospitalisation, all statistically significant except for age 31–50 (*p* = 0.071), unemployed participants (*p* = 0.071), students (*p* = 0.655) and participants with underweight (*p* = 0.157). Self-reported total sleep hours also decreased significantly in all almost all groups except for students (*p* = 0.180) and participants that were underweight (*p* = 0.714), however, this could be explained by small sample size in these categories. No significant differences in sleep quantity or quality could be observed between groups for motive of admission before or during hospitalisation (all > 0.05).

It was shown that age, income, room type, length of hospital stay, smoking or recreational drug intake, or BMI had no significant influence on the patients’ sleep quality at admission or during the hospital stay (all *p* > 0.05). Patients’ sleep quality scores at admission were higher in patients admitted to medical units (Z = −4.144, *p* < 0.001) and a higher PSQI score could be demonstrated in female patients (Z = −3.284, *p* = 0.001). Also, a significant difference between groups at admission could be observed for patients’ working situation, with a better sleep quality for employed patients (H(3) = 9.736, *p* = 0.021). However, these differences between groups were not maintained during hospitalisation. Patients’ sleep quality scores during hospitalisation were higher in those with only primary education than in those with education at the secondary or university level (H(2) = 6.246, *p* = 0.044), whereas there was no difference found between these groups at admission. This difference could also be observed in reported sleep hours during hospitalisation (H(2) = 6.833, *p* = 0.033). Having 2 or more comorbidities was associated with a significant difference in sleep quality both at admission (Z = −3.441, *p* = 0.001) and during hospitalisation (Z = −2.731, *p* = 0.006). Patients taking sedative-hypnotic medication had poorer sleep quality both before (Z = −2.285, *p* = 0.022) and during hospitalisation (Z = −6.092, *p* < 0.001) compared to those who didn’t. These differences were not shown for sleep duration.

Finally, correlation with the Spearman’s Rho test showed evidence that higher physical activity measured with the IPAQ questionnaire led to better sleep quality during hospitalisation (R = −0.148, *p* = 0.012). However, differences were not significant at admission, nor were they significant for sleep quantity. No other differences between groups for both sleep quality (Table 3) or quantity (Table 4) were statistically significant.

**Table 3:** Effects PSQI global scores and sleep quantity.

	Sleep quality (PSQI global scores)					
	At admission			During hospitalisation		
	n	Mean	SD	n	Mean	SD
**Age**						
< 30	8	6.25	4.37	8	13.13	5.41
31–50	44	7.84	4.14	44	10.59	3.69
51–70	140	8.56	4.59	140	10.94	3.82
> 70	106	9.20	4.44	106	11.96	4.27
Test and *p*-value**	H (3) = 5.208, *P* = 0.157			H (3) = 6.528, *P* = 0.089		
**Unit**						
Surgical unit	139	7.49	4.42	139	11.15	4.03
Medical unit	158	9.65	4.30	158	11.51	4.02
Test and *p*-value**	Z = −4.144, *P* < 0.001			Z = −0.591, *P* = 0.555		
**Gender**						
Male	173	7.86	4.32	173	11.15	3.80
Female	124	9.67	4.54	124	11.49	4.35
Test and *p*-value**	Z = -3.284, *P* < 0.001			Z = 0–565, *P* = 0.572		
**Hospital stay**						
< 7 days?	105	8.76	4.44	105	10.9	4.11
> 7 days?	156	8.57	4.56	156	11.37	3.94
Test and *p*-value**	Z = 0.502, *P* = 0.616			Z = −0.990, *P* = 0.322		
**Educational level**						
Primary	161	8.65	4.69	161	11.85	4.19
Secondary	98	8.66	4.31	98	10.58	3.86
University	29	8.00	3.85	29	11.00	3.64
Test and *p*-value**	H (2) = 0.724, *P* = 0.696			H (2) = 6.246, *P* = 0.044		
**Working situation**						
Employed	84	7.50	4.38	84	10.57	3.75
Unemployed	32	9.66	4.67	32	11.34	4.27
Retired	176	8.91	4.44	176	11.63	4.08
Student	2	9.50	0.71	2	11.50	7.78
Test and *p*-value**	H (3) = 9.736, *P* = 0.021			H (3) = 3.974, *P* = 0.264		
**Income (euros/month)**						
0–500	26	8.15	3.83	26	11.35	3.52
501–1000	92	8.84	4.60	92	10.85	4.42
1001–2000	103	8.17	4.65	103	11.16	4.23
> 2000	30	8.83	3.76	30	11.33	3.69
Test and *p*-value**	H (3) = 1.514, *P* = 0.679			H (3) = 0.32, *P* = 0.956		
**Room type**						
Single	46	7.26	4.55	46	10.65	3.76
Double	252	8.87	4.44	252	11.43	4.08
Test and p-value**	Z = -1.945, *P* = 0.052			Z = −1.19, *P* = 0.231		
**BMI**						
< 18.5	3	13.00	4.58	3	12.33	4.04
18.5 to < 25	99	8.28	4.23	99	11.78	4.04
25 to < 30	108	8.38	4.82	108	11.10	3.96
30 or more	85	9.01	4.28	85	10.85	4.13
Test and *p*-value**	H (3) = 1.514, *P* = 0.642			H (3) = 2.909, *P* = 0.406		
**Comorbidities**						
Single	73	7.14	3.84	73	10.15	3.93
Multimborbidity	183	9.28	4.52	183	11.6	4.08
Test and *p*-value**	Z = −3.441, *P* = 0.001			Z = −2.731, *P* = 0.006		
**Sedative hypnotic**						
Intake	126	9.34	4.55	126	13.04	3.82
No intake	172	8.08	4.37	172	10.04	3.71
Test and *p*-value**	Z = −2.285, *P* = 0.022			Z = −6.092, *P* < 0.001		

**Student’s *t* test or ANOVA.

**Table 4:** Effects sleep quantity (in hours).

	Sleep quantity (h)					
	At admission			During hospitalisation		
	n	Mean	SD	n	Mean	SD
**Age**						
< 30	9	6.92	1.33	9	5.48	1.61
31–50	46	6.03	1.36	46	5.54	1.39
51–70	159	6.16	1.66	159	5.33	1.55
> 70	124	6.48	1.72	124	5.26	1.67
Test and *p*-value**	H (3) = 3.183, *P* = 0.364			H (3) = 1.586, *P* = 0.663		
**Unit**						
Surgical unit	160	6.28	1.51	160	5.42	1.32
Medical unit	177	6.28	1.75	177	5.25	1.77
Test and *p*-value**	Z = −0.264, P = 0.792			Z = −0.639, P = 0.523		
**Gender**						
Male	196	6.32	1.59	196	5.37	1.44
Female	141	6.21	1.71	141	5.29	1.76
Test and *p*-value**	Z = −0.645, *P* = 0.519			Z = −0.554, *P* = 0.579		
**Hospital stay**						
< 7 days?	123	6.29	1.65	123	5.32	1.65
> 7 days?	174	6.24	1.64	174	5.37	1.48
Test and *p*-value**	Z = −0.393, *P* = 0.694			Z = −0.152, *P* = 0.879		
**Educational level**						
Primary	184	6.35	1.71	184	5.18	1.65
Secondary	111	6.16	1.60	111	5.62	1.54
University	33	6.39	1.16	33	5.24	1.19
Test and *p*-value**	H (2) = 0.871, *P* = 0.647			H (2) = 6.833, *P* = 0.033		
**Working situation**						
Employed	97	6.21	1.35	97	5.43	1.49
Unemployed	35	5.89	1.68	35	5.30	1.50
Retired	200	6.38	1.75	200	5.31	1.63
Student	2	8.00	0.71	2	6.40	1.27
Test and *p*-value**	H (3) = 4.859, *P* = 0.182			H (3) = 1.646, *P* = 0.649		
**Income (euro/month)**						
0–500	29	6.38	1.49	29	5.34	1.40
501–1000	101	6.43	1.76	101	5.54	1.62
1001–2000	122	6.04	1.65	122	5.28	1.75
> 2000	32	6.81	1.55	32	5.48	1.31
Test and *p*-value** lePara>	H (3) = 4.756, *P* = 0.191			H (3) = 1.597, *P* = 0.660		
**Room type**						
Single	52	6.46	1.62	52	5.51	1.41
Double	286	6.25	1.64	286	5.31	1.60
Test and *p*-value**	Z = −0.807, *P* = 0.42			Z = −1.364, *P* = 0.173		
**BMI**						
< 18.5	5	5.50	2.29	5	5.60	0.82
18.5 to < 25	114	6.34	1.63	114	5.30	1.54
25 to < 30	122	6.43	1.72	122	5.35	1.53
30 or more	94	6.11	1.51	94	5.39	1.71
Test and *p*-value**	H (3) = 2.407, *P* = 0.492			H (3) = 0.200, *P* = 0.978		
**Comorbidities**						
Single	78	6.49	1.56	78	5.48	1.44
Multimborbidity	213	6.17	1.66	210	5.21	1.63
Test and *p*-value**	Z = −1.274, *P* = 0.203			Z = −1.311, *P* = 0.190		
**Sedative hypnotic**						
Intake	141	6.18	1.80	141	5.40	1.5
No intake	197	6.34	1.50	197	5.29	1.57
Test and *p*-value**	Z = −1.039, *P* = 0.299			Z = −0.493, *P* = 0.622		

**Student’s *t* test or ANOVA.

## Discussion

To our knowledge, this nationwide, multicentre study is the first to examine subjective sleep quality and quantity in patients of surgical and medical units in 12 public hospitals in Spain. Sleep is necessary for the human being to lead normal activities and maintain good health^36^. Hospitalised patients who are recovering from surgery or illness are supposed to have an even greater need of restorative sleep to achieve full recovery^37^. However, our results demonstrated that sleep quality in patients admitted to hospitals of the public national health system experienced poorer sleep quality during their hospitalisation compared to at home, and slept almost one hour less than before hospitalisation. This suggests that the experience of hospitalisation has a negative effect on both self-reported sleep quality and sleep duration. These findings are in line with numerous other studies reporting poor sleep quality and quantity in hospitalised patients in countries such as China^21^ , the Netherlands^22^, Turkey^38–40^ Canada^41^ and Germany^42^. Although full comparison with other studies could not be done because of reporting on patients from specific units, single hospital samples, patients with specific conditions, or the use of other sleep quality measurement tools. However, this may be an indication that, despite previous research showing poor sleep quality of hospitalised patients, in general there is still a problem in getting good sleep quality in a hospital. Even if hospital technology and hospital buildings have become more sophisticated^43^ there is still room for improvement in the way they address one of the basic needs of patients during their recovery, such as their sleep. More research is needed to identify factors that help promoting good night-time sleep in hospitals. Therefore, we believe that this study adds information to the existing literature.

Highest PSQI scores during hospitalisation could be observed in the components of sleep latency, sleep duration and sleep efficiency which may suggest a lack of the use of behavioural intervention to improve the sleep experience in hospital^44^. Using the cut-of score of 5 proposed by Buysse et al.^2^, more than three quarters of our included sample had poor sleep quality at admission to the hospital with a mean PSQI score of 8.62. This mean score is higher than in recent studies on sleep quality in the general population in Spain who reported PSQI scores of 5.14^45^ and 5.45^46^ respectively. This higher score may be due to the effects of acute illness before hospitalisation. Also, our data were collected during the COVID-19 pandemic. Although our study did not include COVID-19 patients, there is evidence that COVID-19 outbreak-associated factors correlate with a decrease in sleep quality^46^. These findings were confirmed by a recent study who concluded that both patients with and without an acute COVID-19 infection experienced poor sleep quality and quantity^47^. This would also explain why the results of a study conducted during the COVID-19 period on general Spanish population observed a similar global PSQI score^5,14^ with our study population^48^. During the COVID-19 outbreak an increase of stress, anxiety and depression affected the general population^49,50^. Anxiety and emotional distress have been shown to be negatively correlated to sleep quality in hospitalised^41,51,52^. Stress-induced activation of the hypothalamic–pituitary–adrenal (HPA) axis disrupts sleep, and variations in sleep enhance this activation, causing a vicious cycle of stress and sleeplessness^53^. Also, quarantine and social isolation may have caused changes in sleep–wake rhythms and a reduction of sleep quality^54^. These factors could have had an even greater impact on hospitalised patients during peak COVID-19 periods due to restrictions on visitors or night-companions, or a higher fear of getting infected with COVID-19 in the hospital^55^. Treatment delay because of the pandemic could also have caused patients to be in a more acute phase of their illness before getting admitted to the hospital.

Our sample was comprised of 83% of patients older than 50 years and 37% older than 70 years. Changes in the normal sleep-cycle and a variety of sleep problems are reported in elderly people and sleep quality is expected to deteriorate with age^56^. Our study found higher PSQI scores as age groups increased, but differences were not found to be significant at admission or during hospitalisation. Having higher educational level was observed to be a protective factor for poor sleep quality and quantity during hospitalisation, however, these results could be related to the age of the participants as secondary education became mandatory in 1964 in Spain^57^. At admission women had poorer sleep quality than men. Taking into account the mean age of our sample, it has been demonstrated that several hormonal factors, such as menopause, and physical changes in women’s life can have impact on their sleep health^58^.

Patients with 2 or more comorbidities were found to have poorer sleep quality which could also be related to higher age, but also because of more physical symptoms. Higher physical activity has also been shown to lead to better sleep quality during hospitalisation and no association was found for BMI which confirms the findings of prior researches^59,60^.

Although a decrease in the consumption of benzodiazepines is reported in Spain^61^, over 40% of our included population regularly took sedative medication at home or in hospital. A negative association at admission and during hospitalisation was found for sedative medication intake and sleep quality. This confirms findings of other studies suggesting that benzodiazepines do not improve sleep quality and cause a significant risk of falls, especially in elderly patients what makes that it should be regularly reviewed whether intake is necessary^62–64^ and the implementation of other non-pharmacological therapies with a long-term effect and fewer side effects should be considered^65^. The length of hospital stay was not associated with differences in sleep quality or quantity in our sample, although due to our inclusion criteria of a minimum length of stay of 4 nights in hospital, short-term effects of hospitalisation on the sleep wake cycle could not be fully explored.

Management of sleep in hospital is challenging as intrinsic sleep disturbances are clinically heterogeneous and complex^38^. Also, although it is observed that sleep is insufficient among patients, several studies reported a lack of the use of standardized sleep assessment tools and sleep promoting interventions in hospital^66^.

### Limitations

This study has some limitations. The dependence on patient recall for their regular sleep time prior to and during hospitalisation is one of the study’s limitations. Reported sleep quality before admission may have been deviated from habitual sleep quality because of the situation of illness of the participant. We used the PSQI to measure sleep quantity and quality, we did not report on sleep dimensions with objective measurements, future research could focus on verifying and comparing our findings with non-subjective sleep evaluation methods such as actigraphy or polysomnography. Also, The PSQI was developed evaluating “usual” sleep of the past month^2^. PSQI scores during hospitalisation could be biased because of a short hospital stay and patients with a hospital stay shorter than 7 days could not have had the opportunity to answer correctly on the 4 answers provided in some components of the PSQI. This could have led to a sub estimation of sleep quality during hospitalisation. However, median length of stay was 8 days and before passing the test to patients, we made a clear statement that this their answers should only reflect on sleep experience during their stay in hospital. Finally, the study counted 13% missing data in the PSQI test. This can be explained by the high workload of the nurses who could not go through every questionnaire or by the acute illness which caused patients to leave some questions unanswered. Although there is no established cut-off from the literature regarding an acceptable percentage of missing data in a data set for valid statistical inference, Enders^67^ stated that a missing rate of 15–20% was common.

## Conclusion

This study demonstrated poor sleep quality in patients admitted to hospitals of the public health system in Spain. Significant differences between sleep quality at admission and during hospitalisation in most of the components and global score of the PSQI were found, and inpatients slept a mean difference of 56 min less. A lower educational level, two or more morbidities and the intake of sedative hypnotic medication were shown to be associated with poorer sleep in hospital. However, a higher physical activity level before hospitalisation may function as a protective factor for poor sleep quality in hospital. These results may contribute to drawing patients’, and professionals’ and policymakers’ attention on the importance of sleep both before and during hospitalisation taking into account sociodemographic and clinical variables of the patient.

Future research should focus on disturbing factors causing this sleep quality and quantity decrease in order to draw attention of healthcare workers and design evidence-based interventions that improve the sleep experience in the hospital. Additionally, the positive impact of physical activity on hospitalised patients has to be studies further. Also, however the use of benzodiazepines is decreasing, a high prevalence of dispense is maintained, especially in population over 65 years, to lower this rate integral actions are needed.

## Data Availability

The datasets generated during and/or analysed during the current study are available from the corresponding author on reasonable request.
